# Meningitis after elective intracranial surgery: a systematic review and meta-analysis of prevalence

**DOI:** 10.1186/s40001-023-01141-3

**Published:** 2023-06-08

**Authors:** Rafał Chojak, Marta Koźba-Gosztyła, Magdalena Gaik, Marta Madej, Aleksandra Majerska, Oskar Soczyński, Bogdan Czapiga

**Affiliations:** 1 Faculty of Medicine, Wroclaw Medical University, Ludwika Pasteura 1, 50-367 Wrocław, Poland; 2 Department of Neurosurgery, 4th Military Hospital in Wroclaw, Wrocław, Poland; 3 Department of Nervous System Diseases, Faculty of Health Sciences, Wroclaw Medical University, Wrocław, Poland

**Keywords:** Aseptic meningitis, Bacterial meningitis, Craniotomy, Elective intracranial surgery, Intracranial procedures, Tumor resection, Microvascular decompression, Aneurysm clipping, Prevalence

## Abstract

Meningitis is a potential complication of elective intracranial surgery (EIS). The prevalence of meningitis after EIS varies greatly in the literature. The objective of this study was to estimate the overall pooled prevalence of meningitis following EIS. Four databases (PubMed, Scopus, Web of Science, and Embase) were searched to identify relevant studies. Meta-analyses of proportions were used to combine data. Cochran's *Q* and *I*^2^ statistics were used to assess and quantify heterogeneity. Additionally, several subgroup analyses were conducted to investigate the source of heterogeneity and examine differences in the prevalence based on variables such as geographical regions, income level, and meningitis type. The meta-analysis included 83 studies (30 959 patients) from 26 countries. The overall pooled prevalence of meningitis after EIS was 1.6% (95% CI 1.1–2.1), with high heterogeneity present (*I*^2^ = 88%). The pooled prevalence in low- to middle-income countries and high-income countries was 2.7% (95% CI 1.6–4.1) and 1.2% (95% CI 0.8–1.7), respectively. Studies that reported only aseptic meningitis had a pooled prevalence of 3.2% (95% CI 1.3–5.8). The pooled prevalence was 2.8% (95% CI 1.5–4.5) in studies that reported only bacterial meningitis. Similar prevalence rates of meningitis were observed in the subgroups of tumor resection, microvascular decompression, and aneurysm clipping. Meningitis is a rare but not exceptional complication following EIS, with an estimated prevalence of 1.6%.

**Supplementary Information:**

The online version contains supplementary material available at 10.1186/s40001-023-01141-3.

## Introduction

Meningitis is a potential complication of elective intracranial surgery (EIS). Post-EIS meningitis can be either bacterial or aseptic [1, 2]. Symptoms of both types of meningitis are similar and include fever, neck stiffness, decreased level of consciousness, and headache [3–8]. However, the symptoms of bacterial meningitis tend to be more severe than those of aseptic meningitis [3]. Meningitis without any of these symptoms is very unlikely [3, 4]. A diagnosis of meningitis is made based on clinical symptoms and/or positive cerebrospinal fluid (CSF) bacterial cultures [9, 10].

Postoperative bacterial meningitis can be caused by either Gram-positive or Gram-negative bacteria [3, 6, 11]. It is a serious and potentially life-threatening complication that can lead to severe complications [4]. Therefore, prompt and appropriate medical treatment is essential [4, 12].

Aseptic meningitis refers to inflammation of the meninges without evidence of bacterial infection [3]; it is most likely caused by an inflammatory reaction to the surgical procedure and red blood cell breakdown products [13, 14], although the exact mechanism of postoperative aseptic meningitis is not fully understood. Other possible causes of postoperative meningitis include drug-induced aseptic meningitis [15, 16].

The prevalence of meningitis after EIS varies greatly in the literature, ranging from 0.1 [17] to almost 10% [18] in large-series studies. Moreover, studies reporting on meningitis after EIS are often single-center with small sample sizes, which limits the generalizability of the findings.

The main objective of this meta-analysis was to estimate the overall pooled prevalence of meningitis following EIS. We also hypothesized that the prevalence of post-EIS meningitis would differ based on geographical location and income levels (as in the case of community-acquired meningitis [19, 20]). Additionally, our second hypothesis was that the prevalence of post-EIS meningitis would be similar between bacterial and aseptic meningitis.

## Methods

### Overview

The meta-analysis was conducted in accordance with the preferred reporting items for systematic reviews (PRISMA) guidelines and recommendations [21].

### Search strategy

We performed an electronic search of English-language articles published between January 2000 and September 2022. We searched PubMed, Scopus, Web of Science, and Embase to identify relevant studies. The key terms were associated with common elective intracranial procedures and pathologies, in particular: craniotomy, tumor resection, microvascular decompression (MVD), vascular lesion resection, aneurysm clipping, and meningitis. The search syntax is presented in Additional file 1: Appendix 1. Three researchers (MM, AM, and OS) independently screened all titles and abstracts for their eligibility for inclusion. The full texts of articles that were potentially relevant for the meta-analysis were retrieved for a detailed eligibility assessment according to the selection criteria. Any discrepancies during the selection and extraction processes were resolved by discussion and consensus. Furthermore, we hand-searched the bibliographies of included articles and related reviews to identify additional studies relevant to the meta-analysis.

### Selection criteria

We included studies that reported meningitis following intracranial surgery. The exclusion criteria were: (1) emergency surgery; (2) endonasal surgery; (3) surgery for central nervous system infections, temporal lobe epilepsy, congenital malformations, shunt and Ommaya reservoir-placement, and cranioplasty; (4) stereotactic and functional neurosurgery; (5) studies with less than 50 patients; (6) pediatric studies; (7) nationwide and statewide datasets, reviews, case studies, conference abstracts, animal studies, and letters to the editor.

All studies that missed key data on the number of meningitis cases, contained non-extractable data on the number of meningitis cases after surgery, and had potentially overlapping data were identified and excluded.

### Data extraction

Three reviewers (MM, AM, and OS) independently extracted data from included articles into a spreadsheet using Microsoft Excel (2010; Microsoft Corporation, Redmond, WA, USA). We recorded: (a) the last name of the first author; (b) the year of publication; (c) the country in which the study was conducted; (d) enrollment dates; (e) the age of the patients; (f) the number of patients; (g) the number of cases of post-operative meningitis; and (h) the reported type of meningitis (aseptic, bacterial, both, or none).

### Statistical analysis

The primary outcome of interest was the proportion of meningitis cases after EIS. The meta-analysis was conducted to calculate the overall pooled prevalence. We used the DerSimonian and Laird random-effects model [22] since we assumed high between-study heterogeneity. A Freeman–Tukey double arcsine transformation was used to stabilize the variance [23]. To evaluate the heterogeneity, we used Cochran’s Q test and the *I*^2^ statistic, which represents the percentage of total variation across studies. A Cochran's Q *P*-value of 0.10 indicates significant heterogeneity. The *I*^2^ statistic value of greater than 50% suggests substantial heterogeneity [24]. To assess how much the effect size varies, we calculated the prediction interval for the primary outcome and subgroup analyses.

In order to test our hypothesis, we performed a predefined subgroup analysis by income level according to the World Bank income classification (low- and middle-income countries [LMICs] and high-income countries [HICs]), continent, and WHO regions (African Region (AFR), Region of the Americas (AMR), South-East Asia Region (SEAR), European Region (EUR), Eastern Mediterranean Region (EMR), and Western Pacific Region (WPR)) under the WHO classification (https://www.who.int/countries), country, and type of meningitis. We also did a post hoc analysis by type of procedure. A subgroup analysis and univariate meta-regression by publication year, sample size, quality of included studies, and each covariate included in the subgroup analysis were further investigated for association with the pooled estimates and as potential sources of heterogeneity. The subgroup analyses were performed when data from at least two studies were available. A leave-one-out sensitivity analysis was performed to assess the robustness of the results. Publication bias was visualized with a funnel plot and evaluated using Egger's regression test. A value of *P* less than 0.05 was considered significant. The differences between two subgroups were considered insignificant if their confidence intervals (CI) overlapped. Results are presented in a forest plot with a 95% CI and 95% prediction intervals. All analyses were done in RStudio (version 1.3.1093).

### Level of evidence

Study quality was assessed using modified Oxford Centre for Evidence-Based Medicine recommendations [25]. Studies were rated from 1 (low risk of bias or high quality) to 5 (high risk of bias or low quality). Randomized clinical trials were rated 1; nonrandomized clinical trials and prospective comparative studies were rated 2; case–control studies and retrospective cohort studies were rated 3; case series and cross-sectional studies were rated 4; and case reports were rated 5.

## Results

### Search results

**Fig. 1:**
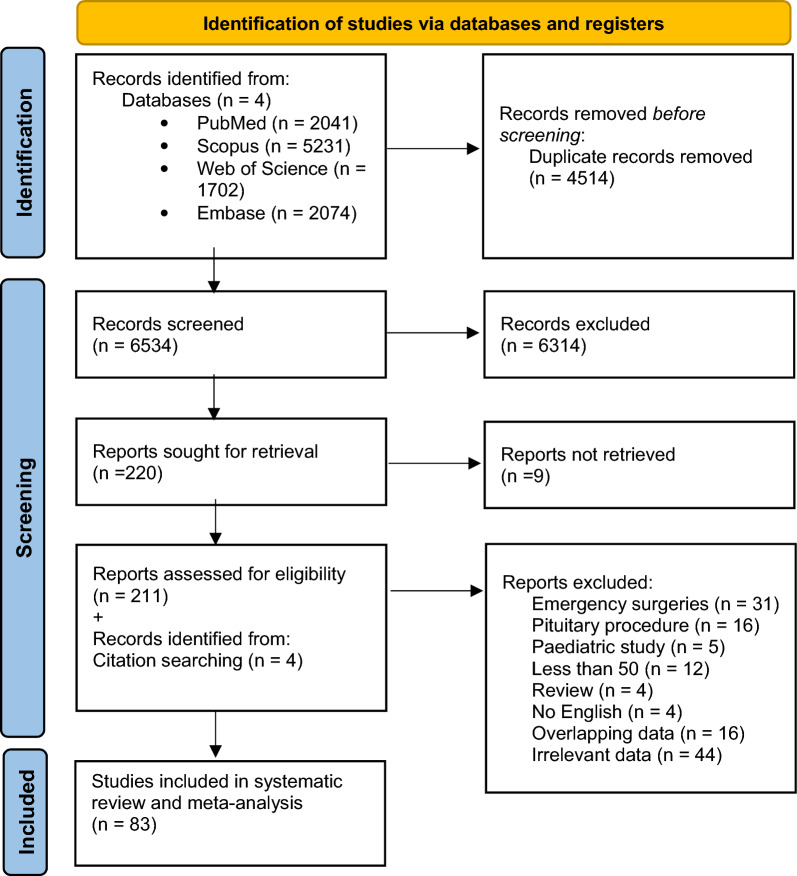
Flowchart showing search strategy. *From:* Page MJ, McKenzie JE, Bossuyt PM, Boutron I, Hoffmann TC, Mulrow CD, et al. The PRISMA 2020 statement: an updated guideline for reporting systematic reviews. BMJ 2021;372:n71. https://doi.org/10.1136/bmj.n71. For more information, visit: http://www.prisma-statement.org/

Database searching yielded 11 048 results, of which 4514 were duplicates. All titles and abstracts of 6534 articles were screened for potentially relevant data. Of these, 220 articles were qualified for full-text evaluation. Additionally, four articles were identified during the hand-searching of bibliographies of relevant articles and included in the meta-analysis. Finally, 83 articles [17, 18, 26–69, 69–106] from 26 different countries were included in the meta-analysis. The process of study identification is shown in Fig. 1.

Thirty-one of the included studies were conducted in Asia, 26 in Europe, 22 in North America, 2 in South America, and 2 in Africa. The most common neurosurgical procedures described in the included articles were tumor resection, followed by microvascular decompression and aneurysm clipping. Using modified Oxford Centre for Evidence-Based Medicine recommendations, we have identified 74 studies (89.2%) as level 4 (case series and cross-sectional studies) and 9 studies (10.18%) as level 2 (nonrandomized clinical trials and prospective comparative studies). The characteristics of the included studies are presented in Additional file 2: Appendix 2.

### Overall prevalence of meningitis after EIS

Among the 30 959 patients (from 83 studies) included in the meta-analysis, 616 experienced meningitis. The prevalence of meningitis after EIS reported in the included studies ranged from 0.0% to 15.3%. The overall pooled estimate was 1.6% (95% CI 1.1–2.1). The 95% prediction interval was 0.0–7.4. The results of the pooled analysis are presented in Table 1.

**Table 1:** Overall prevalence of meningitis after EIS and subgroup analysis by WHO region, continent, and income

Variable	No. of studies (no. of patients)	Pooled prevalence (95% CI)	*I*^2^ (%)	*P*-value (Cochran’s Q)	95% prediction interval
Overall	83 (30,959)	1.6 (1.1–2.1)	88	< 0.01	0.0–7.4
WPR	25 (6421)	2.4 (1.1–4.2)	92	< 0.01	0.0–15.3
AMR	24 (10,462)	0.9 (0.4–1.6)	84	< 0.01	0.0–4.9
EUR	27 (7730)	1.4 (0.8–2.3)	82	< 0.01	0.0–6.8
EMR	3 (202)	3.9 (0.2–10.7)	68	0.04	–
SEAR	3 (5993)	1.8 (1.2–2.6)	24	0.27	0.0–12.7
Asia	31 (12,566)	2.5 (1.4–3.7)	90	< 0.01	0.0–11.4
Europe	26 (7679)	1.4 (0.7–2.2)	82	< 0.01	0.0–6.7
North America	22 (10,022)	0.9 (0.3–1.5)	85	< 0.01	0.0–4.9
South America	2 (440)	1.5 (0.0–4.7)	73	0.05	–
Africa	2 (252)	1.2 (0.1–3.1)	0	0.90	–
LMICs	24 (9899)	2.7 (1.6–4.1)	88	< 0.01	0.0–11.4
HICs	59 (21,060)	1.2 (0.8–1.7)	83	< 0.01	0.0–5.6

WPR: Western Pacific Region; AMR: Americas Region; EUR: European Region; EMR: Eastern Mediterranean Region; SEAR: South-East Asian Region; LMIC: low- and middle-income countries; HIC: high-income countries

### Meningitis prevalence after EIS by geographic location

**Fig. 2:**
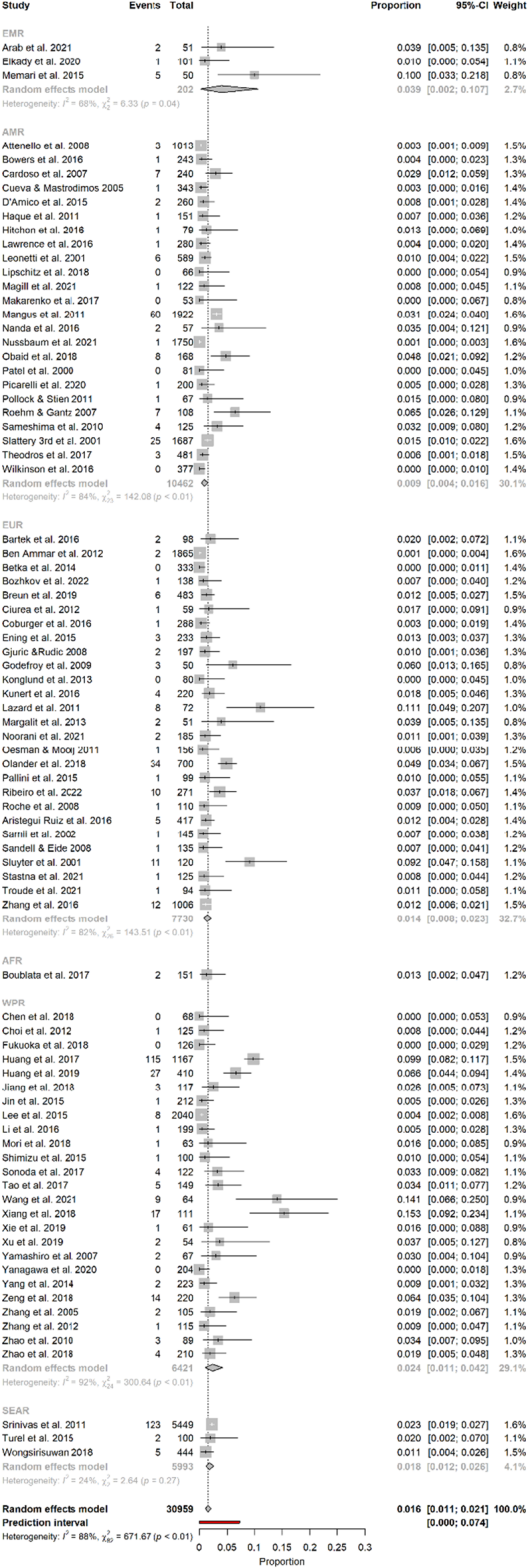
Forest plot of the prevalence of meningitis after EIS by WHO region. WPR: Western Pacific Region; AMR: Region of Americas; EUR: European Region; EMR: Eastern Mediterranean Region; SEAR: South-East Asian Region; AFR: African Region

The highest pooled prevalence of meningitis after EIS was in EMR (3.9%), followed by WPR (2.4%). The AMR had the lowest pooled prevalence of meningitis after EIS (0.9%). The results of the pooled analysis by WHO region are presented in Table 1 and Fig. 2. In the subgroup analysis by continent, the highest prevalence of meningitis after EIS was in Asia (2.5%), followed by South America (1.5%). The results for estimates by continent are shown in Table 1 and Additional file 3: Appendix 3. Estimates by country are shown in Additional file 4: Appendix 4.

### Meningitis prevalence after EIS by income

We identified 24 papers from LMICs and 59 from HICs reporting meningitis after EIS. The pooled prevalence was 2.7% and 1.2% in the LMICs and HICs, respectively. The results for estimates by income level are shown in Table 1 and Additional file 5: Appendix 5.

### Prevalence of meningitis after EIS by type of meningitis

Most studies did not report data on meningitis types, and nine studies reported no cases of meningitis after EIS. Eleven studies reported data on aseptic meningitis. The pooled prevalence in the subgroup was 3.2%. Bacterial meningitis was reported in nine studies. The pooled prevalence was 2.8%. Data on both aseptic meningitis and bacterial meningitis were reported in seven studies, with an estimated pooled prevalence of 2.9%. Table 2 and Additional file 6: Appendix 6 show the results of the pooled analysis by type of meningitis.

**Table 2:** Subgroup analysis of meningitis after EIS by type of meningitis and type of surgery

Variable	No. of studies (no. Of patients)	Pooled prevalence (%) (95% CI)	*I*^2^ (%)	*P*-value (Cochran’s Q)	95% prediction interval
Aseptic	11 (2258)	3.2 (1.3–5.8)	88	< 0.01	0.0–16.1
Bacterial	9 (7380)	2.8 (1.5–4.5)	81	< 0.01	0.0–9.2
Aseptic + Bacterial	7 (7114)	2.9 (0.8–6.0)	97	< 0.01	0.0–18.3
TR	55 (17,662)	1.9 (1.2–2.6)	89	< 0.01	0.0–9.0
VS	30 (12,988)	2.0 (1.1–3.0)	92	< 0.01	0.0–10.1
SBM	9 (717)	1.3 (0.4–2.5)	8	0.37	0.1–3.3
HGG	5 (1708)	0.7 (0.1–1.8)	61	0.04	0.0–5.6
MVD	21 (5701)	1.2 (0.5–2.0)	77	< 0.01	0.0–5.9
TN	13 (2505)	1.6 (0.5–3.0)	80	< 0.01	0.0–8.9
HS	5 (2614)	0.6 (0.1–1.4)	40	0.16	0.0–3.4
AN	5 (2086)	0.4 (0.0–2.1)	68	0.01	0.0–9.0

TR: tumor resection; VS: vestibular schwannoma; SBM: skull base meningioma; HGG: high-grade glioma; MVD: microvascular decompression; TN: trigeminal neuralgia; HS: hemifacial spasm; AN: aneurysm clipping

### Meningitis prevalence after EIS by procedure type

Fifty-five studies reported on meningitis after tumor resection. Meningitis after microvascular decompression and aneurysm clipping were reported in 21 and 5 studies, respectively. The pooled prevalence of meningitis after tumor resection, MVD, and aneurysm clipping was 1.9%, 1.2%, and 0.4%, respectively. Table 2 and Additional file 7: Appendix 7 show the results of a subgroup analysis based on the type of procedure.

### Between-study heterogeneity and publication bias

**Fig. 3:**
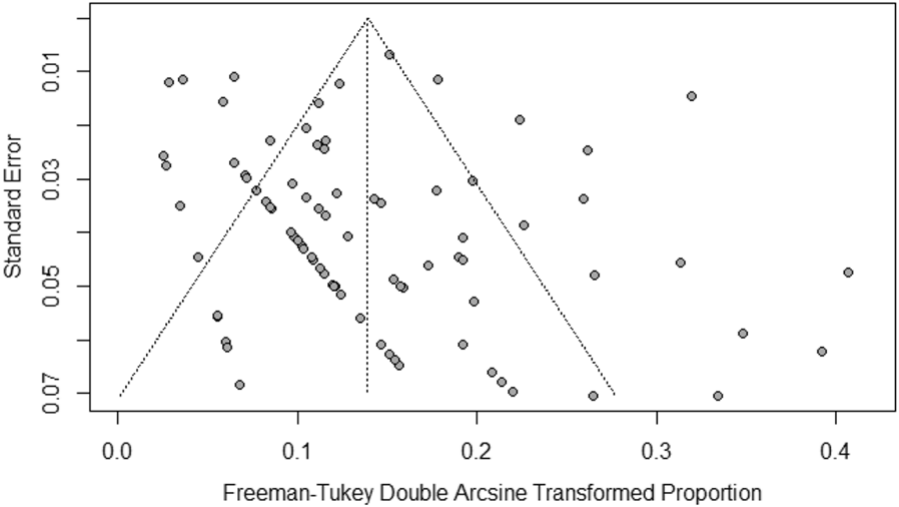
Funnel plot of included studies

We found no significant publication bias in the included studies on the prevalence of meningitis after EIS, as shown by the funnel plot (Fig. 3) and the Egger’s linear regression test of funnel plot asymmetry *(P* = 0.2228). An influential analysis showed that the study by Huang et al. [18] contributed the most to the overall prevalence of meningitis after EIS and between-study heterogeneity. After omitting this study, the overall prevalence and heterogeneity decreased to 1.45% and *I*^2^ = 83.5%, respectively (Additional file 8: Appendix 8). The results of univariate meta-regression showed that neither publication year (*P* = 0.82; *R*^2^ = 0%) nor sample size (*P* = 0.40; *R*^2^ = 0%) were associated with the prevalence of meningitis after EIS; these covariates could not explain heterogeneity either. The results of univariate meta-regression for covariates included in a subgroup analysis were as follows: WHO region (*P* = 0.22; *R*^2^ = 0%), continent (*P* = 0.11; *R*^2^ = 0.73%), country (*P* = 0.15; *R*^2^ = 14.29%), income (*P* = 0.007; *R*^2^ = 13.19%), surgery type (*P* = 0.62; *R*^2^ = 0%), and type of meningitis (*P* = 0.0004; *R*^2^ = 6.11%).

## Discussion

This systematic review and meta-analysis analyzed data from 83 studies representing 26 countries and including 30 959 patients. The objective was to estimate the prevalence of meningitis following EIS. The results showed an overall prevalence of 1.6%. The prevalence of post-EIS meningitis was found to be slightly higher in the subgroup of countries with low to middle-income levels when compared to high-income countries. We did not find evidence of a significant difference in the prevalence of post-EIS meningitis between different WHO regions and continents. Furthermore, there were no significant differences in the prevalence of post-EIS meningitis between bacterial and aseptic meningitis. The included studies were highly heterogeneous, and only part of the heterogeneity was explained by the meta-regression, subgroup, and sensitivity analyses.

The highest prevalence of postoperative meningitis was reported by Xiang et al. [95] (15.3%) and Wang et al. [92] (14.1%). The authors reported aseptic meningitis after MVD (for trigeminal neuralgia) and jugular foramen tumor surgery, respectively. In both studies, the authors did not provide a possible explanation for the high prevalence of postoperative meningitis. Some of the risk factors reported in the literature include CSF leakage, perioperative steroid use, older age, external ventricular or lumbar drainage, repeat operations, surgery duration, and increased intraoperative blood loss [11, 36, 107–109]. The estimated prevalence of post-EIS meningitis in this study is comparable to the overall rate of nosocomial meningitis reported by Korinek et al. [110]. In their study, over 21% of procedures were emergent. Although they did not find emergency procedures to be a risk factor for postoperative meningitis, other studies have reported it [107, 109].

In the study of vestibular schwannomas by Huang et al. [18], just a tiny portion (3.48%) of the cerebrospinal fluid (CSF) samples collected from suspected meningitis patients tested positive for bacterial meningitis, indicating that the majority of cases were likely aseptic. This study's findings showed similar rates of aseptic and bacterial meningitis, however, the limited reporting of CSF culture results in most studies made it challenging to conduct a comprehensive analysis of the prevalence of each type of meningitis. The causes of aseptic meningitis might sometimes be misunderstood. The term "aseptic" refers to any meningitis with negative CSF bacterial cultures. And it might be caused by either infectious (viral, parasitic, or fungal) or non-infectious causes [10]. Reaction to heme breakdown products released during surgery appears to be the most likely cause of post-neurosurgical aseptic meningitis [6]. This could explain the high proportion of aseptic meningitis reported in several large-series studies of tumor resection [18, 83] since these procedures are often associated with substantial blood loss [111]. The duration of the operation and the size of the tumor are risk factors for meningitis after tumor surgery [49, 107], and both might be associated with increased blood loss.

This study has some limitations. First, database searching was limited by language restrictions. This might have resulted in omitting potentially relevant non-English studies, introducing language bias. Second, database searching was limited to studies published after 2000. This was done to estimate the relatively current status of the prevalence of meningitis after EIS. Third, the exclusion criteria applied limit the results’ generalizability to all elective intracranial procedures. Therefore, its important to consider the methods used in this study when interpreting the presented results, as they do not cover certain elective intracranial procedures (e.g., shunt placement or functional neurosurgery). Fourth, the majority of the studies included were retrospective case series, which have inherent limitations. These studies often report the mean or median age rather than an age range. Hence, it is possible that some of the studies included patients who were under 18 years old. Fifth, due to the large number of screened results, there is a possibility that some studies might have been omitted. However, the impact of potentially omitted studies should be minimized by the relatively large number of included studies. Sixth, the small number of included studies was from EMR, SEAR, AFR, South America, and Africa; thus, this might have affected the estimates for these subgroups as well as overall estimates. Finally, we could not control for many possible sources of heterogeneity (risk factors for postoperative meningitis, patient characteristics, scrub techniques, antibiotic prophylaxis, and patients' pre- and post-operative management) due to the lack of data.

Despite these limits, this is the first systematic review and meta-analysis to estimate a pooled prevalence of meningitis after EIS. Subgroup and sensitivity analyses, as well as meta-regression, were performed to examine the various factors likely to affect our results and account for between-study variance. Finally, since the presence of high heterogeneity may be of concern, we provided prediction intervals, as recommended by Migliavaca et al. [112].

## Conclusions

Meningitis is a rare but not exceptional complication of elective intracranial surgery, with an overall estimated pooled prevalence of 1.6% and a prediction interval of 0–7.4%. Improving the quality of observational studies reporting on meningitis, in particular in terms of reporting meningitis type and CSF culture results, might be beneficial for future evaluation of this issue.

## Supplementary Information

**Additional file 1: Appendix 1.** Literature search strategy.

**Additional file 2: Appendix 2.** Characteristics of included studies.

**Additional file 3:** **Appendix 3.** Forest plot of the prevalence of meningitis after elective intracranial surgery (EIS) by continents.

**Additional file 4: Appendix 4.** Subgroup analysis of meningitis after elective intracranial surgery (EIS) by country.

**Additional file 5: Appendix 5.** Forest plot of the prevalence of meningitis after elective intracranial surgery (EIS) by income.

**Additional file 6: Appendix 6.** Forest plot of the prevalence of meningitis after elective intracranial surgery (EIS) by type of meningitis.

**Additional file 7: Appendix 7.** Forest plot of the prevalence of meningitis after elective intracranial surgery (EIS) by type of surgery.

**Additional file 8: Appendix 8.** An influential analysis (Random effects model).

## Data Availability

Data collected for the study and the code used to perform the meta-analyses, meta-regression, and sensitivity analyses can be made available upon reasonable request to the corresponding author.
