# Supplementary material for: Meningitis after elective intracranial surgery: a systematic review and meta-analysis of prevalence
Source: Eur J Med Res. 2023 Jun 8;28:184. doi: 10.1186/s40001-023-01141-3 (PMC10249328; doi:10.1186/s40001-023-01141-3)
Supplement: Supplementary file 1 — Additional file 1: Appendix 1. Literature search strategy. [file 40001_2023_1141_MOESM1_ESM.docx]

**Title: Meningitis after elective intracranial surgery: a systematic review and meta-analysis of prevalence**

**Authors:** Rafał Chojak ^1^, Marta Koźba-Gosztyła ^2^ , Magdalena Gaik ^1^, Marta Madej ^1^, Aleksandra Majerska ^1^, Oskar Soczyński ^1^, Bogdan Czapiga ^2,3^

^1^ Faculty of Medicine, Wroclaw Medical University, Wroclaw, Poland

^2^ Department of Neurosurgery, 4th Military Hospital in Wroclaw, Wroclaw, Poland

^3^ Department of Nervous System Diseases, Faculty of Health Sciences, Wroclaw Medical University, Wroclaw, Poland

Corresponding author:
Rafał Chojak
E-mail: [rafalchojak@gmail.com](mailto:rafalchojak@gmail.com)

| PubMed |
| --- |
| **Concept 1**  **("Brain Neoplasms"[MeSH Terms] OR "Intracranial Aneurysm"[MeSH Terms] OR "Trigeminal Neuralgia"[MeSH Terms] OR "Central Nervous System Vascular Malformations"[MeSH Terms] OR Tumor*[Title/Abstract] OR tumour*[Title/Abstract] OR neoplasm*[Title/Abstract] OR lesion*[Title/Abstract] OR cancer*[Title/Abstract] OR glio*[Title/Abstract] OR Astrocytoma*[Title/Abstract] OR meningioma*[Title/Abstract] OR schwannoma*[Title/Abstract] OR neuroma*[Title/Abstract] OR neurocytoma*[Title/Abstract] OR neurofibroma*[Title/Abstract] OR hemangioblastoma*[Title/Abstract] OR chondroma*[Title/Abstract] OR osteoma*[Title/Abstract] OR ependymal*[Title/Abstract] OR ependymoma*[Title/Abstract] OR lymphoma*[Title/Abstract] OR neuroblastoma*[Title/Abstract] OR medulloblastoma*[Title/Abstract] OR hemangiopericytoma*[Title/Abstract] OR ganglio*[Title/Abstract] OR pinealocytoma*[Title/Abstract] OR aneurysm*[Title/Abstract] OR aneurism*[Title/Abstract] OR "trigeminal neuralgia"[Title/Abstract] OR neuralgia[Title/Abstract] OR malformation*[Title/Abstract] OR nerve*[Title/Abstract] OR "vascular lesion"[Title/Abstract])**  **Concept 2**  **("Neurosurgical Procedures"[Mesh] OR Craniotom*[Title/Abstract] OR neurosurg*[Title/Abstract] OR surger*[Title/Abstract] OR resection*[Title/Abstract] OR debulking*[Title/Abstract] OR decompression*[Title/Abstract] OR operat*[Title/Abstract] OR excision*[Title/Abstract] OR clipping*[Title/Abstract] OR ectom*[Title/Abstract])**  **Concept 3**  **(meningitis[mesh] OR meningitis[Text Word] OR "meningeal infection"[Text Word] OR "meningeal inflammation"[Text Word])**  **Concept 4**  (2000:2022[pdat]) |

**Appendix 1.** Literature Search Strategy.

| Scopus |
| --- |
| **Concept 1**  TITLE-ABS-KEY(tumor* OR tumour* OR neoplasm* OR lesion* OR cancer* OR glio* OR astrocytoma* OR meningioma* OR schwannoma* OR neuroma* OR neurocytoma* OR neurofibroma* OR hemangioblastoma* OR chondroma* OR osteoma* OR ependymal* OR ependymoma* OR lymphoma* OR neuroblastoma* OR medulloblastoma* OR hemangiopericytoma* OR ganglio* OR pinealocytoma* OR aneurysm* OR aneurism* OR "trigeminal neuralgia" OR neuralgia OR malformation* OR nerve* OR "vascular lesion")  **Concept 2**  TITLE-ABS-KEY(craniotom* OR neurosurg* OR surger* OR resection* OR debulking* OR decompression* OR operat* OR excision* OR clipping* OR ectom*)  **Concept 3**  TITLE-ABS-KEY(meningitis OR "meningeal infection" OR "meningeal inflammation")  **Concept 4**  LIMIT-TO ( PUBYEAR,2022) OR LIMIT-TO ( PUBYEAR,2021) OR LIMIT-TO ( PUBYEAR,2020) OR LIMIT-TO ( PUBYEAR,2019) OR LIMIT-TO ( PUBYEAR,2018) OR LIMIT-TO ( PUBYEAR,2017) OR LIMIT-TO ( PUBYEAR,2016) OR LIMIT-TO ( PUBYEAR,2015) OR LIMIT-TO ( PUBYEAR,2014) OR LIMIT-TO ( PUBYEAR,2013) OR LIMIT-TO ( PUBYEAR,2012) OR LIMIT-TO ( PUBYEAR,2011) OR LIMIT-TO ( PUBYEAR,2010) OR LIMIT-TO ( PUBYEAR,2009) OR LIMIT-TO ( PUBYEAR,2008) OR LIMIT-TO ( PUBYEAR,2007) OR LIMIT-TO ( PUBYEAR,2006) OR LIMIT-TO ( PUBYEAR,2005) OR LIMIT-TO ( PUBYEAR,2004) OR LIMIT-TO ( PUBYEAR,2003) OR LIMIT-TO ( PUBYEAR,2002) OR LIMIT-TO ( PUBYEAR,2001) OR LIMIT-TO ( PUBYEAR,2000) ) |

| Web of Science |
| --- |
| **Concept 1**  TS=(Tumor OR tumour OR neoplasm OR lesion OR cancer OR glioma OR glioblastoma OR astroglioma OR glial OR Astrocytoma OR meningioma OR schwannoma OR neuroma OR neurocytoma OR neurofibroma OR hemangioblastoma OR chondroma OR osteoma OR ependymal OR ependymoma OR lymphoma OR neuroblastoma OR medulloblastoma OR hemangiopericytoma OR ganglioma OR pinealocytoma OR aneurysm OR aneurism OR “trigeminal neuralgia” OR neuralgia OR malformation OR nerve OR “vascular lesion”)  **Concept 2**  TS=(Craniotomy OR neurosurgery OR surgery OR resection OR debulking OR decompression OR operation OR excision OR clipping OR ectomy)  **Concept 3**  TS=(meningitis OR "meningeal infection" OR "meningeal inflammation")  **Concept 4**  2000-01-01 - 2022-09-01 |

| Embase |
| --- |
| **Concept 1**  (tumor:ti,ab,kw OR tumour:ti,ab,kw OR neoplasm:ti,ab,kw OR lesion:ti,ab,kw OR cancer:ti,ab,kw OR glioma:ti,ab,kw OR glioblastoma:ti,ab,kw OR astroglioma:ti,ab,kw OR astrocytoma:ti,ab,kw OR meningioma:ti,ab,kw OR schwannoma:ti,ab,kw OR neuroma:ti,ab,kw OR neurocytoma:ti,ab,kw OR neurofibroma:ti,ab,kw OR hemangioblastoma:ti,ab,kw OR chondroma:ti,ab,kw OR osteoma:ti,ab,kw OR ependymal:ti,ab,kw OR ependymoma:ti,ab,kw OR lymphoma:ti,ab,kw OR neuroblastoma:ti,ab,kw OR medulloblastoma:ti,ab,kw OR hemangiopericytoma:ti,ab,kw OR ganglio:ti,ab,kw OR pinealocytoma:ti,ab,kw OR aneurysm:ti,ab,kw OR aneurism:ti,ab,kw OR 'trigeminal neuralgia':ti,ab,kw OR neuralgia:ti,ab,kw OR malformation:ti,ab,kw OR nerve:ti,ab,kw OR 'vascular lesion':ti,ab,kw)  **Concept 2**  (craniotomy:ti,ab,kw OR neurosurgery:ti,ab,kw OR surgery:ti,ab,kw OR resection:ti,ab,kw OR debulking:ti,ab,kw OR decompression:ti,ab,kw OR operation:ti,ab,kw OR excision:ti,ab,kw OR clipping:ti,ab,kw OR ectomy:ti,ab,kw)  **Concept 3**  (meningitis:ti,ab,kw OR 'meningeal infection':ti,ab,kw OR 'meningeal inflammation':ti,ab,kw)  **Concept 4**  [2000-2022]/py |
