# Supplementary material for: Meningitis after elective intracranial surgery: a systematic review and meta-analysis of prevalence
Source: Eur J Med Res. 2023 Jun 8;28:184. doi: 10.1186/s40001-023-01141-3 (PMC10249328; doi:10.1186/s40001-023-01141-3)
Supplement: Supplementary file 2 — Additional file 2: Appendix 2. Characteristics of included studies. [file 40001_2023_1141_MOESM2_ESM.docx]

**Title: Meningitis after elective intracranial surgery: a systematic review and meta-analysis of prevalence**

**Authors:** Rafał Chojak ^1^, Marta Koźba-Gosztyła ^2^ , Magdalena Gaik ^1^, Marta Madej ^1^, Aleksandra Majerska ^1^, Oskar Soczyński ^1^, Bogdan Czapiga ^2,3^

^1^ Faculty of Medicine, Wroclaw Medical University, Wroclaw, Poland

^2^ Department of Neurosurgery, 4th Military Hospital in Wroclaw, Wroclaw, Poland

^3^ Department of Nervous System Diseases, Faculty of Health Sciences, Wroclaw Medical University, Wroclaw, Poland

Corresponding author:
Rafał Chojak
E-mail: [rafalchojak@gmail.com](mailto:rafalchojak@gmail.com)

**Appendix 2.** Characteristics of included studies

| **Study** | **Recruitment period** | **Country** | **Level of evidence** | **WHO Region** | **Income level** | **N** | **Events** | **Age** | **% Female** | **Meningitis Type** | **Surgery Type** |
| --- | --- | --- | --- | --- | --- | --- | --- | --- | --- | --- | --- |
| Xiang et al. 2018 | 2014-2016 | China | 2 | WPR | LMIC | 111 | 17 | 60,6 | 55,0 | aseptic | MVD |
| Lazard et al. 2011 | 2007 | France | 2 | EUR | HIC | 72 | 8 | 50,0 | 44,4 | aseptic | TR |
| Memari et al. 2015 | NA | Iran | 4 | EMR | LMIC | 50 | 5 | 49,0 | 45,0 | bacterial | TR |
| Huang et al. 2017 | 1999-2013 | China | 4 | WPR | LMIC | 1167 | 115 | 47,5 | 54,2 | bacterial&aseptic | TR |
| Sluyter et al. 2001 | 1986-1999 | Netherlands | 4 | EUR | HIC | 120 | 11 | 49,0 | 51,7 | bacterial | TR |
| Huang et al. 2019 | 2015-2018 | China | 4 | WPR | LMIC | 410 | 27 | 50,0 | 58,0 | aseptic | TR |
| Roehm & Gantz 2007 | 1980-2004 | US | 4 | AMR | HIC | 108 | 7 | 70,4 | ND | ND | TR |
| Zeng et al. 2018 | 2015-2016 | China | 2 | WPR | LMIC | 220 | 14 | 56,0 | 57,7 | aseptic | MVD |
| Godefroy et al. 2009 | 2000-2005 | Netherlands | 4 | EUR | HIC | 50 | 3 | 49,0 | 66,0 | bacterial | TR |
| Olander et al. 2018 | 1988-2014 | Sweden | 4 | EUR | HIC | 700 | 34 | 53,0 | 52,9 | ND | TR |
| Obaid et al. 2018 | 2007-2013 | Canada | 4 | AMR | HIC | 168 | 8 | 51,9 | 53,2 | bacterial | TR |
| Margalit et al. 2013 | 2003-2010 | Israel | 4 | EUR | HIC | 51 | 2 | 57.1 | 74,5 | bacterial&aseptic | TR |
| Nanda et al. 2016 | 1990-2014 | US | 4 | AMR | HIC | 57 | 2 | 58,8 | 59,6 | ND | TR |
| Zhao et al. 2010 | 2005-2008 | China | 4 | WPR | LMIC | 89 | 3 | 48,2 | 57,3 | bacterial | TR |
| Tao et al. 2017 | 2008-2017 | China | 4 | WPR | LMIC | 149 | 5 | 51,0 | 75,2 | bacterial&aseptic | TR |
| Sameshima et al. 2010 | 1998-2007 | US | 4 | AMR | HIC | 125 | 4 | 52,0 | 45,6 | ND | TR |
| Yamashiro et al. 2007 | 2002-2003 | Japan | 2 | WPR | HIC | 67 | 2 | 61,3 | 71,6 | ND | AN |
| Cardoso et al. 2007 | 1990-2005 | Brazil | 4 | AMR | LMIC | 240 | 7 | ND | ND | ND | TR |
| Srinivas et al. 2011 | 2001-2007 | India | 4 | SEAR | LMIC | 5449 | 123 | ND | ND | bacterial | VA |
| Turel et al. 2015 | 2009-2012 | India | 4 | SEAR | LMIC | 100 | 2 | 44,2 | 35,0 | ND | TR |
| Zhang et al. 2005 | 1997-2002 | China | 4 | WPR | LMIC | 105 | 2 | 46,8 | 61,0 | bacterial | TR |
| Zhao et al. 2018 | 2010-2015 | China | 4 | WPR | LMIC | 210 | 4 | 57,0 | 60,5 | ND | MVD |
| Kunert et al. 2016 | 1990-2011 | Poland | 4 | EUR | HIC | 220 | 4 | 18-74 | 60,9 | ND | TR |
| Xie et al. 2019 | 2011-2016 | China | 4 | WPR | LMIC | 61 | 1 | 34,4 | 56,5 | ND | VL |
| Mori et al. 2018 | 2005-2014 | Japan | 4 | WPR | HIC | 63 | 1 | 64,0 | 65,1 | ND | AN |
| Pollock & Stien 2011 | 1999-2009 | US | 2 | AMR | HIC | 67 | 1 | 74,0 | 53,7 | aseptic | MVD |
| Slattery 3rd et al. 2001 | 1987-1997 | US | 4 | AMR | HIC | 1687 | 25 | 49,8 | 51,0 | bacterial&aseptic | TR |
| Aristegui Ruiz et al. 2016 | 1994-2014 | Spain | 4 | EUR | HIC | 417 | 5 | 49,8 | 50,1 | ND | TR |
| Zhang et al. 2016 | 1990-2006 | France | 4 | EUR | HIC | 1006 | 12 | 55,6 | 53,1 | bacterial | TR |
| Leonetti et al. 2001 | 1988-1999 | US | 4 | AMR | HIC | 589 | 6 | 56,4 | 54,2 | ND | TR |
| Pallini et al. 2015 | 1984-2010 | Italy | 4 | EUR | HIC | 99 | 1 | 57,0 | 64,6 | ND | TR |
| Shimizu et al. 2015 | 2012-2014 | Japan | 2 | WPR | HIC | 100 | 1 | 58,9 | 76,0 | ND | MVD |
| Elkady et al. 2020 | 2012-2017 | Egypt | 4 | EMR | LMIC | 101 | 1 | 55,1 | 62,3 | ND | TR |
| Roche et al. 2008 | 1991-2001 | France | 4 | EUR | HIC | 110 | 1 | 50,1 | 60,0 | ND | TR |
| Yang et al. 2014 | 2006-2011 | China | 4 | WPR | LMIC | 223 | 2 | 59,5 | 52,5 | ND | MVD |
| Zhang et al. 2012 | 2001-2010 | China | 4 | WPR | LMIC | 115 | 1 | 46,4 | 56,5 | ND | TR |
| Choi et al. 2012 | 2007-2010 | Korea | 4 | WPR | HIC | 125 | 1 | 57,8 | 68,8 | ND | AN |
| Sandell & Eide 2008 | 1999-2005 | Norway | 4 | EUR | HIC | 135 | 1 | 65,0 | 54,8 | aseptic | MVD |
| Haque et al. 2011 | 1998-2009 | US | 4 | AMR | HIC | 151 | 1 | 49,0 | ND | ND | TR |
| Oesman & Mooij 2011 | 1983-2003 | Netherlands | 4 | EUR | HIC | 156 | 1 | 58,3 | 57,7 | ND | MVD |
| Theodros et al. 2017 | 1998-2015 | US | 4 | AMR | HIC | 481 | 3 | 50,0 | 70,6 | ND | MVD |
| Picarelli et al. 2020 | 2010-2016 | Brazil | 2 | AMR | LMIC | 200 | 1 | 56,1 | 55,0 | ND | TR |
| Lee et al. 2015 | 1998-2013 | Korea | 4 | WPR | HIC | 2040 | 8 | 50,0 | 70,5 | bacterial&aseptic | MVD |
| Attenello et al. 2008 | 1996-2006 | US | 4 | AMR | HIC | 1013 | 3 | 51,0 | 41,0 | ND | TR |
| Cueva & Mastrodimos 2005 | 1996-2004 | US | 4 | AMR | HIC | 343 | 1 | ND | ND | bacterial | TR |
| Ben Ammar et al. 2012 | 1987-2009 | Italy | 4 | EUR | HIC | 1865 | 2 | 50,4 | 52,5 | ND | TR |
| Betka et al. 2014 | 1997-2012 | Czech Republic | 4 | EUR | HIC | 333 | 0 | 48,0 | 57,0 | - | TR |
| Yanagawa et al. 2020 | 2014-2018 | Japan | 4 | WPR | HIC | 204 | 0 | 61,0 | 63,7 | - | MVD |
| Konglund et al. 2013 | 2008-2009 | Norway | 2 | EUR | HIC | 80 | 0 | 68,5 | 47,5 | - | TR |
| Patel et al. 2000 | 1997 | US | 4 | AMR | HIC | 81 | 0 | ND | 79,0 | - | AN |
| Lipschitz et al. 2018 | 2007-2017 | US | 4 | AMR | HIC | 66 | 0 | 51,7 | 51,5 | - | TR |
| Stastna et al. 2021 | 2005-2019 | UK | 4 | EUR | HIC | 125 | 1 | 56 | 48 | ND | TR |
| Bozhkov et al. 2022 | 2014-2017 | Germany | 4 | EUR | HIC | 138 | 1 | 55,6 | 50 | ND | TR |
| Ribeiro et al. 2022 | 2018-2019 | Portugal | 4 | EUR | HIC | 271 | 10 | 55,9 | 59,4 | ND | TR |
| Nussbaum et al. 2021 | 1997-2019 | US | 4 | AMR | HIC | 1750 | 1 | 44 | 62 | ND | AN |
| Arab et al. 2021 | NA | Saudi Arabia | 4 | EMR | HIC | 51 | 2 | 48 | 76,5 | ND | TR |
| Troude et al. 2021 | 2000-2018 | France | 4 | EUR | HIC | 94 | 1 | 55 | 88 | ND | TR |
| Wang et al. 2021 | 2011-2020 | China | 4 | WPR | LMIC | 64 | 9 | 43 | 51,6 | ND | TR |
| Magill et al. 2021 | 2014-2018 | US | 4 | AMR | HIC | 122 | 1 | 57 | 70 | ND | TR |
| Noorani et al. 2021 | 1996-2012 | UK | 4 | EUR | HIC | 185 | 2 | 58,5 | 52,4 | A | MVD |
| Fukuoka et al. 2018 | 2003-2015 | Japan | 4 | WPR | HIC | 126 | 0 | 62,1 | 57,9 | - | MVD |
| Jiang et al. 2018 | 2010-2016 | China | 4 | WPR | LMIC | 117 | 3 | 45,8 | 67,5 | ND | MVD |
| Chen et al. 2018 | 2010-2016 | China | 2 | WPR | LMIC | 68 | 0 | 59,3 | 66,2 | - | MVD |
| Xu et al. 2019 | 2013-2017 | China | 4 | WPR | LMIC | 54 | 2 | 53,2 | 72 | ND | TR |
| Breun et al. 2019 | 2005-2016 | Germany | 4 | EUR | HIC | 483 | 6 | 50 | 53 | ND | TR |
| Wongsirisuwan 2018 | 2007-2016 | Thailand | 4 | SEAR | LMIC | 444 | 5 | ND | 67 | A | MVD |
| Makarenko et al. 2017 | 2001-2013 | Canada | 4 | AMR | HIC | 53 | 0 | 57,4 | 73,6 | - | TR |
| Sonoda et al. 2017 | 2013-2015 | Japan | 4 | WPR | HIC | 122 | 4 | 66 | 41 | A | TR |
| Boublata et al. 2017 | 2010-2015 | Africa | 4 | AFR | LMIC | 151 | 2 | 48,2 | 64,9 | ND | TR |
| Lawrence et al. 2016 | 2007-2015 | US | 4 | AME | HIC | 280 | 1 | 57,4 | 63,9 | A | MVD |
| Coburger et al. 2016 | 2000-2014 | Germany | 4 | EUR | HIC | 288 | 1 | 39 | ND | ND | TR |
| Li et al. 2016 | 1993-2003 | China | 4 | WPR | LMIC | 199 | 1 | 46.3 | 68.8 | ND | TR |
| Hitchon et al. 2016 | 2001-20015 | US | 4 | AMR | HIC | 79 | 1 | 57 | 58.2 | ND | MVD |
| Bowers et al. 2016 | 2000-2012 | US | 4 | AMR | HIC | 243 | 1 | 49.3 | 46.5 | ND | TR |
| Wilkinson et al. 2016 | 2002-2012 | US | 4 | AMR | HIC | 377 | 0 | ND | ND | ND | TR |
| Bartek et al. 2016a | 1994-2013 | Norway | 4 | EUR | HIC | 98 | 2 | 61 | 65 | AB | MVD |
| D’Amico et al. 2015 | 2000-2012 | US | 4 | AMR | HIC | 260 | 2 | ND | ND | ND | TR |
| Jin et al. 2015 | 2009-2012 | China | 4 | WPR | LMIC | 212 | 1 | 45.6 | 43.4 | A | MVD |
| Ening etal. 2015 | 2006-2011 | Germany | 4 | EUR | HIC | 233 | 3 | 62 | 49.8 | ND | TR |
| Mangus et al. 2011 | 1970-2010 | US | 4 | AMR | HIC | 1922 | 60 | ND | ND | AB | TR |
| Ciurea et al. 2012 | 1991-2008 | Romania | 4 | EUR | HIC | 59 | 1 | 52 | 59.3 | ND | TR |
| Gjuric&Rudic 2008 | ND | Croatia | 4 | EUR | HIC | 197 | 2 | 51 | ND | ND | TR |
| Samii et al. 2002 | 1980-1998 | Germany | 4 | EUR | HIC | 145 | 1 | 54.5 | 62.2 | ND | MVD |

**Abbreviations:**

HIC = high-income countries; LMIC = low- and middle-income countries; AMR = Region of Americas; EMR = Eastern Mediterranean Region, EUR = European Region; SEAR = South-East Asian Region; WPR = Western Pacific Region; AFR = African Region; TR = Tumor resection; MVD = Microvascular decompression; AN = Aneurysm clipping; VA = various; ND = no data
