# Supplementary material for: Meningitis after elective intracranial surgery: a systematic review and meta-analysis of prevalence
Source: Eur J Med Res. 2023 Jun 8;28:184. doi: 10.1186/s40001-023-01141-3 (PMC10249328; doi:10.1186/s40001-023-01141-3)
Supplement: Supplementary file 4 — Additional file 4: Appendix 4. Subgroup analysis of meningitis after elective intracranial surgery (EIS) by country. [file 40001_2023_1141_MOESM4_ESM.docx]

**Title: Meningitis after elective intracranial surgery: a systematic review and meta-analysis of prevalence**

**Authors:** Rafał Chojak ^1^, Marta Koźba-Gosztyła ^2^ , Magdalena Gaik ^1^, Marta Madej ^1^, Aleksandra Majerska ^1^, Oskar Soczyński ^1^, Bogdan Czapiga ^2,3^

^1^ Faculty of Medicine, Wroclaw Medical University, Wroclaw, Poland

^2^ Department of Neurosurgery, 4th Military Hospital in Wroclaw, Wroclaw, Poland

^3^ Department of Nervous System Diseases, Faculty of Health Sciences, Wroclaw Medical University, Wroclaw, Poland

Corresponding author:
Rafał Chojak
E-mail: [rafalchojak@gmail.com](mailto:rafalchojak@gmail.com)

**Appendix 4.** Subgroup analysis of meningitis after elective intracranial surgery (EIS) by Country.

| Variable | **No. Of Studies (no. Of patients)** | **Pooled Prevalence (%) (95% CI)** | **I2** | **P-value (Cochran’s Q)** |
| --- | --- | --- | --- | --- |
| United States | 20 (9801) | 0.8 (0.3-1.4) | 85% | <0.01 |
| China | 17 (3574) | 3.4 (1.7-5.6) | 88% | <0.01 |
| Japan | 6 (682) | 0.9 (0.0-2.6) | 59% | 0.03 |
| Germany | 5 (1287) | 0.9 (0.4-1.5) | 0% | 0.76 |
| France | 4 (1282) | 2.3 (0.2-6.0) | 80% | <0.01 |
| Norway | 3 (313) | 0.7 (0.0-2.2) | 0% | 0.39 |
| Netherlands | 3 (326) | 4.3 (0.1-12.7) | 85% | <0.01 |
| Canada | 2 (221) | 1.9 (0.0-8.9) | 75% | 0.04 |
| United Kingdom | 2 (310) | 1.0 (0.1-2.5) | 0% | 0.89 |
| Brazil | 2 (440) | 1.5 (0.0-4.7) | 73% | 0.05 |
| India | 2 (5549) | 2.0 (1.7-2.4) | 0% | 0.90 |
| Italy | 2 (1964) | 0.2 (0.0-1.8) | 63% | 0.10 |
| Korea | 2 (2165) | 0.3 (0.1-0.6) | 0% | 0.35 |
