# Supplementary material for: Meningitis after elective intracranial surgery: a systematic review and meta-analysis of prevalence
Source: Eur J Med Res. 2023 Jun 8;28:184. doi: 10.1186/s40001-023-01141-3 (PMC10249328; doi:10.1186/s40001-023-01141-3)

**Title: Meningitis after elective intracranial surgery: a systematic review and meta-analysis of prevalence**

**Authors:** Rafał Chojak ^1^, Marta Koźba-Gosztyła ^2^ , Magdalena Gaik ^1^, Marta Madej ^1^, Aleksandra Majerska ^1^, Oskar Soczyński ^1^, Bogdan Czapiga ^2,3^

^1^ Faculty of Medicine, Wroclaw Medical University, Wroclaw, Poland

^2^ Department of Neurosurgery, 4th Military Hospital in Wroclaw, Wroclaw, Poland

^3^ Department of Nervous System Diseases, Faculty of Health Sciences, Wroclaw Medical University, Wroclaw, Poland

Corresponding author:
Rafał Chojak
E-mail: [rafalchojak@gmail.com](mailto:rafalchojak@gmail.com)

**Appendix 5.** Forest plot of the prevalence of meningitis after elective intracranial surgery (EIS) by Income. Abbreviations: HIC = high-income countries; LMIC = low- and middle-income countries


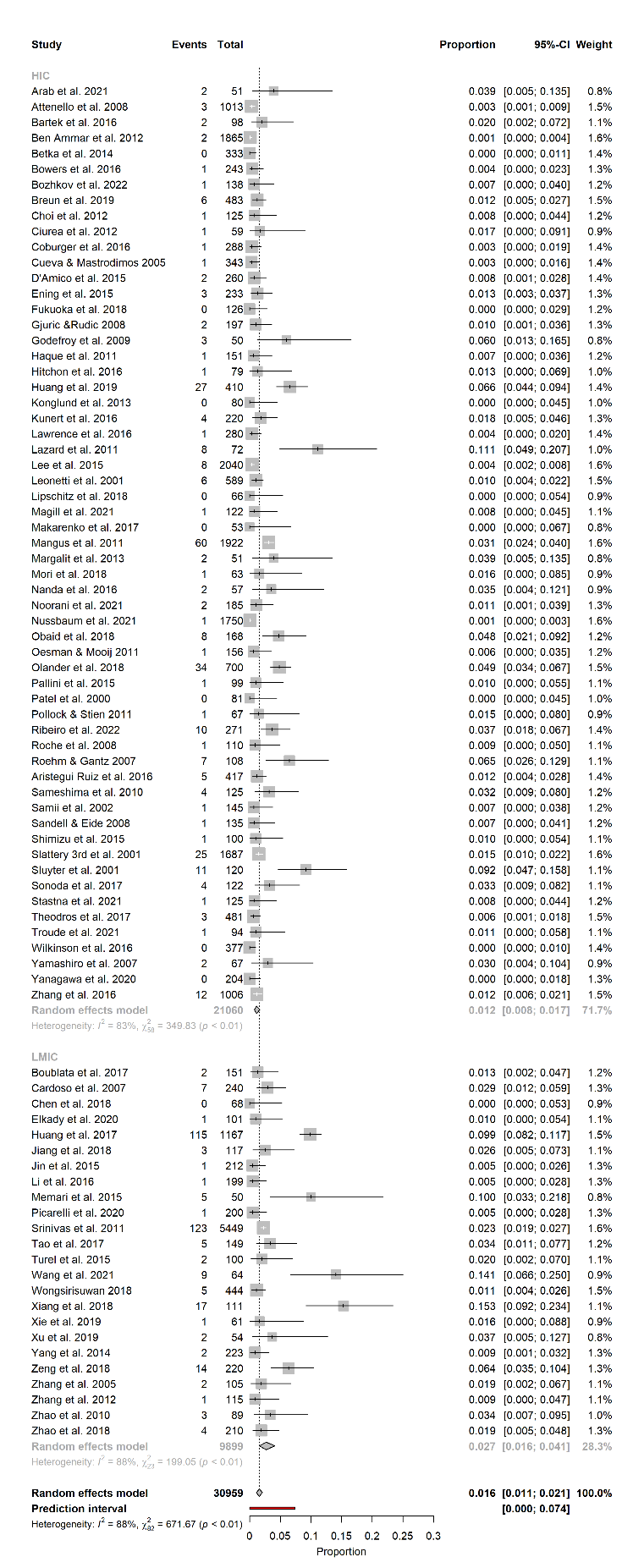

Supplement: Supplementary file 5 — Additional file 5: Appendix 5. Forest plot of the prevalence of meningitis after elective intracranial surgery (EIS) by income. [file 40001_2023_1141_MOESM5_ESM.docx]
