# Supplementary material for: Meningitis after elective intracranial surgery: a systematic review and meta-analysis of prevalence
Source: Eur J Med Res. 2023 Jun 8;28:184. doi: 10.1186/s40001-023-01141-3 (PMC10249328; doi:10.1186/s40001-023-01141-3)
Supplement: Supplementary file 8 — Additional file 8: Appendix 8. An influential analysis (Random effects model). [file 40001_2023_1141_MOESM8_ESM.docx]

**Title: Meningitis after elective intracranial surgery: a systematic review and meta-analysis of prevalence**

**Authors:** Rafał Chojak ^1^, Marta Koźba-Gosztyła ^2^ , Magdalena Gaik ^1^, Marta Madej ^1^, Aleksandra Majerska ^1^, Oskar Soczyński ^1^, Bogdan Czapiga ^2,3^

^1^ Faculty of Medicine, Wroclaw Medical University, Wroclaw, Poland

^2^ Department of Neurosurgery, 4th Military Hospital in Wroclaw, Wroclaw, Poland

^3^ Department of Nervous System Diseases, Faculty of Health Sciences, Wroclaw Medical University, Wroclaw, Poland

Corresponding author:
Rafał Chojak
E-mail: [rafalchojak@gmail.com](mailto:rafalchojak@gmail.com)

**Appendix 8.** An influential analysis (Random effects model).

proportion 95%-CI p-value tau^2 tau I^2

Omitting Arab et al. 2021 0.0156 [0.0110; 0.0207] 0.0050 0.0710 87.9%

Omitting Attenello et al. 2008 0.0160 [0.0114; 0.0213] 0.0051 0.0711 87.6%

Omitting Bartek et al. 2016 0.0156 [0.0111; 0.0208] 0.0051 0.0711 87.9%

Omitting Ben Ammar et al. 2012 0.0161 [0.0115; 0.0212] 0.0048 0.0693 86.7%

Omitting Betka et al. 2014 0.0161 [0.0115; 0.0213] 0.0050 0.0707 87.7%

Omitting Boublata et al. 2017 0.0157 [0.0112; 0.0209] 0.0051 0.0712 87.9%

Omitting Bowers et al. 2016 0.0159 [0.0113; 0.0211] 0.0051 0.0712 87.9%

Omitting Bozhkov et al. 2022 0.0158 [0.0112; 0.0210] 0.0051 0.0712 87.9%

Omitting Breun et al. 2019 0.0158 [0.0112; 0.0210] 0.0051 0.0716 87.9%

Omitting Cardoso et al. 2007 0.0155 [0.0110; 0.0207] 0.0051 0.0712 87.9%

Omitting Chen et al. 2018 0.0159 [0.0114; 0.0211] 0.0050 0.0711 87.9%

Omitting Choi et al. 2012 0.0158 [0.0112; 0.0210] 0.0051 0.0712 87.9%

Omitting Ciurea et al. 2012 0.0157 [0.0111; 0.0209] 0.0051 0.0711 87.9%

Omitting Coburger et al. 2016 0.0159 [0.0113; 0.0212] 0.0051 0.0712 87.9%

Omitting Cueva & Mastrodimos 2005 0.0160 [0.0114; 0.0212] 0.0051 0.0712 87.8%

Omitting D'Amico et al. 2015 0.0158 [0.0112; 0.0211] 0.0051 0.0713 87.9%

Omitting Elkady et al. 2020 0.0158 [0.0112; 0.0210] 0.0051 0.0712 87.9%

Omitting Ening et al. 2015 0.0157 [0.0112; 0.0210] 0.0051 0.0713 87.9%

Omitting Fukuoka et al. 2018 0.0160 [0.0114; 0.0212] 0.0050 0.0710 87.9%

Omitting Gjuric &Rudic 2008 0.0158 [0.0112; 0.0210] 0.0051 0.0713 87.9%

Omitting Godefroy et al. 2009 0.0155 [0.0110; 0.0206] 0.0050 0.0709 87.9%

Omitting Haque et al. 2011 0.0158 [0.0112; 0.0210] 0.0051 0.0712 87.9%

Omitting Hitchon et al. 2016 0.0157 [0.0112; 0.0209] 0.0051 0.0711 87.9%

Omitting Huang et al. 2017 0.0145 [0.0106; 0.0190] 0.0036 0.0604 83.5%

Omitting Huang et al. 2019 0.0151 [0.0107; 0.0202] 0.0049 0.0697 87.4%

Omitting Jiang et al. 2018 0.0156 [0.0110; 0.0208] 0.0051 0.0711 87.9%

Omitting Jin et al. 2015 0.0159 [0.0113; 0.0211] 0.0051 0.0712 87.9%

Omitting Konglund et al. 2013 0.0159 [0.0114; 0.0212] 0.0050 0.0710 87.9%

Omitting Kunert et al. 2016 0.0157 [0.0111; 0.0209] 0.0051 0.0713 87.9%

Omitting Lawrence et al. 2016 0.0159 [0.0113; 0.0212] 0.0051 0.0712 87.9%

Omitting Lazard et al. 2011 0.0152 [0.0107; 0.0202] 0.0049 0.0703 87.7%

Omitting Lee et al. 2015 0.0160 [0.0114; 0.0213] 0.0051 0.0715 87.3%

Omitting Leonetti et al. 2001 0.0158 [0.0112; 0.0211] 0.0051 0.0717 87.9%

Omitting Li et al. 2016 0.0159 [0.0113; 0.0211] 0.0051 0.0712 87.9%

Omitting Lipschitz et al. 2018 0.0159 [0.0113; 0.0211] 0.0050 0.0711 87.9%

Omitting Magill et al. 2021 0.0158 [0.0112; 0.0210] 0.0051 0.0712 87.9%

Omitting Makarenko et al. 2017 0.0159 [0.0113; 0.0211] 0.0051 0.0711 87.9%

Omitting Mangus et al. 2011 0.0155 [0.0109; 0.0208] 0.0052 0.0721 87.5%

Omitting Margalit et al. 2013 0.0156 [0.0110; 0.0207] 0.0050 0.0710 87.9%

Omitting Memari et al. 2015 0.0153 [0.0108; 0.0204] 0.0050 0.0706 87.8%

Omitting Mori et al. 2018 0.0157 [0.0112; 0.0209] 0.0051 0.0711 87.9%

Omitting Nanda et al. 2016 0.0156 [0.0110; 0.0207] 0.0050 0.0710 87.9%

Omitting Noorani et al. 2021 0.0158 [0.0112; 0.0210] 0.0051 0.0713 87.9%

Omitting Nussbaum et al. 2021 0.0161 [0.0116; 0.0212] 0.0047 0.0687 86.5%

Omitting Obaid et al. 2018 0.0154 [0.0109; 0.0205] 0.0050 0.0709 87.8%

Omitting Oesman & Mooij 2011 0.0158 [0.0112; 0.0211] 0.0051 0.0712 87.9%

Omitting Olander et al. 2018 0.0153 [0.0108; 0.0204] 0.0049 0.0703 87.4%

Omitting Pallini et al. 2015 0.0158 [0.0112; 0.0210] 0.0051 0.0712 87.9%

Omitting Patel et al. 2000 0.0159 [0.0114; 0.0212] 0.0050 0.0710 87.9%

Omitting Picarelli et al. 2020 0.0159 [0.0113; 0.0211] 0.0051 0.0712 87.9%

Omitting Pollock & Stien 2011 0.0157 [0.0112; 0.0209] 0.0051 0.0711 87.9%

Omitting Ribeiro et al. 2022 0.0154 [0.0109; 0.0206] 0.0050 0.0711 87.8%

Omitting Roche et al. 2008 0.0158 [0.0112; 0.0210] 0.0051 0.0712 87.9%

Omitting Roehm & Gantz 2007 0.0153 [0.0108; 0.0204] 0.0050 0.0707 87.8%

Omitting Aristegui Ruiz et al. 2016 0.0158 [0.0112; 0.0210] 0.0051 0.0716 87.9%

Omitting Sameshima et al. 2010 0.0155 [0.0110; 0.0207] 0.0051 0.0711 87.9%

Omitting Samii et al. 2002 0.0158 [0.0112; 0.0210] 0.0051 0.0712 87.9%

Omitting Sandell & Eide 2008 0.0158 [0.0112; 0.0210] 0.0051 0.0712 87.9%

Omitting Shimizu et al. 2015 0.0158 [0.0112; 0.0210] 0.0051 0.0712 87.9%

Omitting Slattery 3rd et al. 2001 0.0158 [0.0111; 0.0211] 0.0054 0.0732 87.9%

Omitting Sluyter et al. 2001 0.0151 [0.0107; 0.0202] 0.0049 0.0702 87.6%

Omitting Sonoda et al. 2017 0.0155 [0.0110; 0.0207] 0.0051 0.0711 87.9%

Omitting Srinivas et al. 2011 0.0157 [0.0109; 0.0213] 0.0058 0.0764 87.6%

Omitting Stastna et al. 2021 0.0158 [0.0112; 0.0210] 0.0051 0.0712 87.9%

Omitting Tao et al. 2017 0.0155 [0.0110; 0.0207] 0.0051 0.0711 87.9%

Omitting Theodros et al. 2017 0.0159 [0.0113; 0.0211] 0.0051 0.0715 87.9%

Omitting Troude et al. 2021 0.0158 [0.0112; 0.0210] 0.0051 0.0712 87.9%

Omitting Turel et al. 2015 0.0157 [0.0111; 0.0208] 0.0051 0.0711 87.9%

Omitting Wang et al. 2021 0.0151 [0.0107; 0.0201] 0.0049 0.0700 87.6%

Omitting Wilkinson et al. 2016 0.0161 [0.0115; 0.0213] 0.0050 0.0706 87.7%

Omitting Wongsirisuwan 2018 0.0158 [0.0112; 0.0210] 0.0051 0.0716 87.9%

Omitting Xiang et al. 2018 0.0148 [0.0105; 0.0198] 0.0048 0.0690 87.3%

Omitting Xie et al. 2019 0.0157 [0.0111; 0.0209] 0.0051 0.0711 87.9%

Omitting Xu et al. 2019 0.0156 [0.0110; 0.0207] 0.0050 0.0710 87.9%

Omitting Yamashiro et al. 2007 0.0156 [0.0111; 0.0208] 0.0050 0.0711 87.9%

Omitting Yanagawa et al. 2020 0.0160 [0.0114; 0.0213] 0.0050 0.0709 87.8%

Omitting Yang et al. 2014 0.0158 [0.0112; 0.0210] 0.0051 0.0713 87.9%

Omitting Zeng et al. 2018 0.0152 [0.0108; 0.0203] 0.0050 0.0704 87.7%

Omitting Zhang et al. 2005 0.0157 [0.0111; 0.0208] 0.0051 0.0712 87.9%

Omitting Zhang et al. 2012 0.0158 [0.0112; 0.0210] 0.0051 0.0712 87.9%

Omitting Zhang et al. 2016 0.0158 [0.0112; 0.0211] 0.0052 0.0723 87.9%

Omitting Zhao et al. 2010 0.0155 [0.0110; 0.0207] 0.0050 0.0710 87.9%

Omitting Zhao et al. 2018 0.0157 [0.0111; 0.0209] 0.0051 0.0713 87.9%

Pooled estimate 0.0157 [0.0112; 0.0208] 0.0050 0.0710 87.8%
